# (Cryptand-222)potassium(+) (hydrogensulfido)[5,10,15,20-tetra­kis(2-pival­amido­phen­yl)porphyrinato]ferrate(II)

**DOI:** 10.1107/S1600536809028104

**Published:** 2009-07-22

**Authors:** Mondher Dhifet, Mohamed Salah Belkhiria, Jean-Claude Daran, Habib Nasri

**Affiliations:** a Département de Chimie, Faculté des Sciences de Monastir, Avenue de l’environnement, 5019 Monastir, Tunisia; b Laboratoire de Chimie de Coordination, CNRS UPR 8241, 205 Route de Norbonne, 31077 Toulouse, Cedex 04, France

## Abstract

As part of a systematic investigation for a number of Fe^II^ porphyrin complexes used as biomimetic models for cytochrome P450, crystals of the title compound, [K(C_18_H_36_N_2_O_6_)][Fe^II^(C_64_H_64_N_8_O_4_)(HS)], were prepared. The compound exhibits a non-planar conformation with major ruffling and saddling distortions. The average equatorial iron–pyrrole N atom [Fe—N_p_ = 2.102 (2) Å] bond length and the distance between the Fe^II^ atom and the 24-atom core of the porphyrin ring (Fe—P_C_= 0.558 Å) are typical for high-spin iron(II) penta­coordinate porphyrinates. One of the *tert*-butyl groups in the structure is disordered over two sets with occupancies of 0.84 and 0.16.

## Related literature

For general background to iron(II) porphyrin species and their applications, see: Simonneux & Le Maux (2000 ▶). For a description of the Cambridge Structural Database, see: Allen (2002 ▶). For the synthesis of iron(II) picket fence derivatives, see: Collman *et al. *(1975 ▶); Nasri *et al.* (1987 ▶); Hachem *et al.* (2009 ▶). For related structures, see: English *et al.* (1984 ▶); Nasri *et al.* (2000 ▶). For further details of geometric distortions in related compounds, see: Scheidt & Reed (1981 ▶); Scheidt (2000 ▶); Hu *et al.* (2005 ▶); Jentzen *et al.* (1997 ▶). For comparitive bond lengths, see: Allen *et al.* (1987 ▶). For the treatment of disordered solvent of crystallization, see: Spek (2009 ▶); Stähler *et al.* (2001 ▶); Cox *et al.* (2003 ▶); Mohamed *et al.* (2003 ▶); Athimoolam *et al.* (2005 ▶).
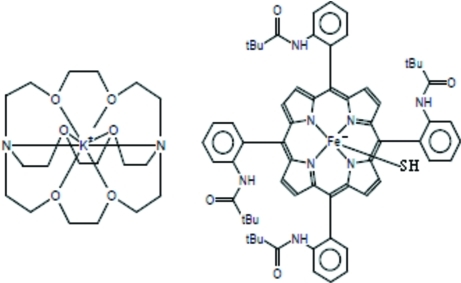


## Experimental

### 

#### Crystal data

[K(C_18_H_36_N_2_O_6_)][Fe(C_64_H_64_N_8_O_4_)(HS)]
*M* *_r_* = 1513.74
Monoclinic, 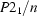

*a* = 17.9327 (7) Å
*b* = 21.5340 (7) Å
*c* = 22.7670 (9) Å
β = 100.611 (2)°
*V* = 8641.4 (6) Å^3^
*Z* = 4
Mo *K*α radiation
μ = 0.31 mm^−1^
*T* = 180 K
0.25 × 0.24 × 0.21 mm

#### Data collection

Bruker APEXII CCD area-detector diffractometer
Absorption correction: multi-scan (*SADABS*; Bruker, 2007 ▶) *T*_min_ = 0.842, *T*_max_ = 0.937
144342 measured reflections
12322 independent reflections
10135 reflections with *I* > 2σ(*I*)
*R*_int_ = 0.048
θ_max_ = 23.2°

#### Refinement

*R*[*F* ^2^ > 2σ(*F* ^2^)] = 0.046
*wR*(*F* ^2^) = 0.128
*S* = 1.05
12322 reflections
965 parameters
9 restraints
H-atom parameters constrained
Δρ_max_ = 0.58 e Å^−3^
Δρ_min_ = −0.45 e Å^−3^

### 

Data collection: *APEX2* (Bruker, 2007 ▶); cell refinement: *SAINT* (Bruker, 2007 ▶); data reduction: *SAINT*; program(s) used to solve structure: *SIR2004* (Burla *et al.*, 2005 ▶); program(s) used to refine structure: *SHELXL97* (Sheldrick, 2008 ▶); molecular graphics: *ORTEPIII* (Burnett & Johnson, 1996 ▶) and *ORTEP-3 for Windows* (Farrugia, 1997 ▶); software used to prepare material for publication: *SHELXL97*.

## Supplementary Material

Crystal structure: contains datablocks I, global. DOI: 10.1107/S1600536809028104/bg2280sup1.cif

Structure factors: contains datablocks I. DOI: 10.1107/S1600536809028104/bg2280Isup2.hkl

Additional supplementary materials:  crystallographic information; 3D view; checkCIF report

## Figures and Tables

**Fig. 1.**
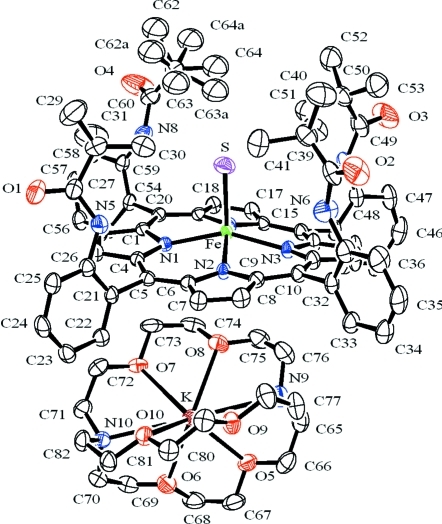
A view of the structure of ion complex [FeII(TpivPP)(SH)]- and the [K(2,2,2,-crypt)]+ counterion showing the atom numbering schem. Displacement ellipsoids are drawn at 50%. The H atoms and the minor disorder tert-butyl group has been omitted for clarity.

**Fig. 2.**
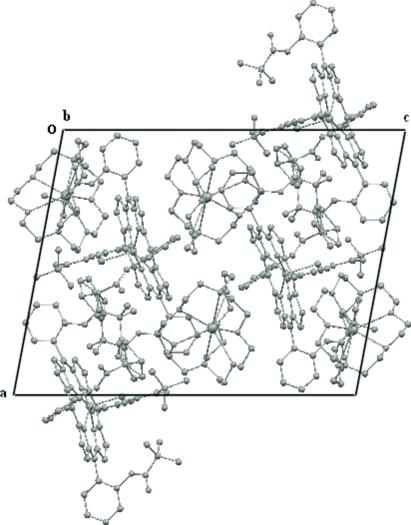
A drawing showing the packing in (I), viewed down the b axis.

## supplementary crystallographic information

### Comment

A large number of iron-thiolate porphyrin complexes have been investigated in order to get more insight into the nature of the electronic and steriochemical properties of cytochromes P450 (Simonneux & Le Maux, 2000). In the Cambridge Structural Database (CSD, Version 5.30 of November 2008; Allen, 2002) there are only three structures of iron(II)-thiolate porphyrinates but no structure of hydrosulfido (SH^-^) iron(II) porphyrinate derivative is reported up to date. We report here the molecular structure of the iron(II) picket fence porphyrin (TpivPP) hydrosulfido species. In the structure of (I), the Fe^2+^ cation is coordinated to the sulfur atom of the SH^-^ axial ligand from the pocket side of the TpivPP porphyrin (Fig. 1). The porphinato core undergoes a significant radial expansion in order to accommodate the high-spin Fe^2+^ cation. This is illustrated by the long Fe—N_p_ and Fe—P_C_ distances shown by these iron(II) high-spin [Fe^II^(Porph)(*X*)]^-^ complexes (*X* = anionic monodentate ligand). The average equatorial Fe—N_p_ distance in (I) [2.102 (2) Å], which is longer than the corresponding Fe^3+^ species [Fe^III^(TAP)(SH)] (English *et al.*, 1984) (TAP = tetrakis(*p*-methoxyphenyl)porphyrinate(2-) [2.015 (2) Å], falls within the range found for five-coordinate high-spin iron(II) porphyrins [2.072- 2.116 Å] (Scheidt & Reed, 1981; Scheidt, 2000; Hu *et al.*, 2005). This is a stereochemical proof that compound (I) is high-spin (S = 2). The Fe—P~C distance [0.7578 Å] is quite longer than those of iron(II) high-spin five-coordinate porphyrines [0.50 – 0.64 Å]. For our model, the axial Fe—S(SH) bond length [2.312 (1) Å] is slightly shorter than those of the three iron(II)-thiolate porphyrinates cited in the literature [2.325 – 2.367 Å]. This distance is longer than the one of the [Fe^III^(TAP)(SH)] derivative [2.298 (3) Å]. It is noteworthy that Fe—SH distance for compound (I) is shorter than the Fe—*S*(thiole) bond length found for iron(II) thiole porphyrin species, *i.e.* for the ion complex [Fe^II^(TpivPP)(NO_2_)(PMS)]^-^ (Nasri *et al.*, 2000) where PMS = pentamethylene sulfide) the Fe—S(PMS) distance is 2.380 (4) Å. The structural decomposition method [NSD] (Jentzen *et al.*, 1997) indicates an important ruffling [41%], a quite high saddling [21%] and a moderate doming [14%] of the porphinato core. The negative charge of the [Fe^II^(TpivPP)(SH)]^-^ anion is balanced by a [K(2,2,2-crypt)]^+^ counterion. The average K—O(2,2,2-crypt) and K—N(2,2,2-crypt) distances [2.827 (2) Å and 3.035 (3) Å respectively] are in agreement with the literature values (Allen *et al.*, 1987). There are no intermolecular or intermolecular hydrogen bonds in the structure of (I). The packing diagram for (I) (Fig.2) is simple. There is no evidence for intermolecular π -π bonding between the faces of the porphyrin cores in compound (I). The absence of the π -π interactions results mainly in the steric restrictions requirements of the pivalamide groups that determine the packing environment.

### Experimental

The reaction sequence leading to the formation of compound (I) is not full understood at present. When a chlorobenzene solution of [Fe^II^(TpivPP)] (Hachem *et al.*, 2009), made *in situ*, is mixed under argon with excess of cryptand-222 and potassium thioacetate (C_2_H_3_OSK) a red-greenish solution was formed. Crystals of (I) were grown by diffusion of hexanes through the chlorobenzene solution.

### Refinement

Due to the diffraction limitation of the crystals of (I) (at 180 K), the data collection was limited to 23.22° in *θ*. Hydrogen atoms were calculated at idealized positions and were refined with 1.2 times the isotropic displacement parameter of the corresponding carbon and nitrogen atoms. The H atom pertaining to the hydrosulfido ligand could not be found in a difference Fourier and was not included in the model.

The *tert-butyl* group of one picket is disordered over two sets. The occupancies of these two positions were refined and then fixed as 0.84 for C62/C63/C64 and 0.16 for C62A/C63A/C64A. The EADP commands in the *SHELXL97* (Sheldrick, 2008) software were used to restrain the parameters of the disordered groups. Some anisotropic displacement ellipsoids of another *tert-butyl *group were rather elongated. This is the case of the anisotropic displacements U22 and U33 of the C29 and C31 carbons of the same *tert-butyl* group.These parameters were restrained to be the same than those of the third CH_3 _group (C30) of the same picket which presents normal ansisotropic displacements for such type of carbon moiety.

At the final stage of refinement, clear evidence of the presence of solvent voids of 241 Å^3^ was obtained (containing approximately 84 electrons). Several trials to find a reasonable model for this were unfruitful. Thus, a correction for diffuse effects due to the inclusion of disordered solvent molecules in the crystal structure was made using the SQUEEZE option in the program *PLATON* (Spek, 2009). The density, the *F(000*) value, the molecular weight and the formula are given without taking into account the results obtained with the SQUEEZE option *PLATON* (Spek, 2009). Similar treatments of disordered solvent molecules have been carried out in this manner (Stähler *et al.* (2001); Cox *et al.* (2003); Mohamed *et al.* (2003); Athimoolam *et al.* (2005).

### Crystal data

**Table:**

[K(C_18_H_36_N_2_O_6_)][Fe(C_64_H_64_N_8_O_4_)(HS)]	*F*(000) = 3216
*M**_r_* = 1513.74	*D*_x_ = 1.164 Mg m^−^^3^
Monoclinic, *P*2_1_/*n*	Mo *K*α radiation, λ = 0.71073 Å
Hall symbol: -P 2yn	Cell parameters from 9881 reflections
*a* = 17.9327 (7) Å	θ = 2.6–23.9°
*b* = 21.5340 (7) Å	µ = 0.31 mm^−^^1^
*c* = 22.7670 (9) Å	*T* = 180 K
β = 100.611 (2)°	Prism, dark purple
*V* = 8641.4 (6) Å^3^	0.25 × 0.24 × 0.21 mm
*Z* = 4	

### Data collection

**Table:**

Bruker APEXII CCD area-detector diffractometer	12322 independent reflections
Radiation source: fine-focus sealed tube	10135 reflections with *I* > 2σ(*I*)
graphite	*R*_int_ = 0.048
φ and ω scans	θ_max_ = 23.2°, θ_min_ = 1.6°
Absorption correction: multi-scan (*SADABS*; Bruker, 2007)	*h* = −17→19
*T*_min_ = 0.842, *T*_max_ = 0.937	*k* = −23→22
144342 measured reflections	*l* = −25→25

### Refinement

**Table:**

Refinement on *F*^2^	Primary atom site location: structure-invariant direct methods
Least-squares matrix: full	Secondary atom site location: difference Fourier map
*R*[*F*^2^ > 2σ(*F*^2^)] = 0.046	Hydrogen site location: inferred from neighbouring sites
*wR*(*F*^2^) = 0.128	H-atom parameters constrained
*S* = 1.05	*w* = 1/[σ^2^(*F*_o_^2^) + (0.0663*P*)^2^ + 5.9377*P*] where *P* = (*F*_o_^2^ + 2*F*_c_^2^)/3
12322 reflections	(Δ/σ)_max_ = 0.048
965 parameters	Δρ_max_ = 0.58 e Å^−^^3^
9 restraints	Δρ_min_ = −0.45 e Å^−^^3^

### Special details

**Table:**

Geometry. All s.u.'s (except the s.u. in the dihedral angle between two l.s. planes) are estimated using the full covariance matrix. The cell s.u.'s are taken into account individually in the estimation of s.u.'s in distances, angles and torsion angles; correlations between s.u.'s in cell parameters are only used when they are defined by crystal symmetry. An approximate (isotropic) treatment of cell s.u.'s is used for estimating s.u.'s involving l.s. planes.
Refinement. Refinement of *F*^2^ against ALL reflections. The weighted *R*-factor *wR* and goodness of fit *S* are based on *F*^2^, conventional *R*-factors *R* are based on *F*, with *F* set to zero for negative *F*^2^. The threshold expression of *F*^2^ > σ(*F*^2^) is used only for calculating *R*-factors(gt) *etc*. and is not relevant to the choice of reflections for refinement. *R*-factors based on *F*^2^ are statistically about twice as large as those based on *F*, and *R*- factors based on ALL data will be even larger.

### Fractional atomic coordinates and isotropic or equivalent isotropic displacement parameters (Å^2^)

**Table:**

	*x*	*y*	*z*	*U*_iso_*/*U*_eq_	Occ. (<1)
Fe	0.042199 (18)	0.071342 (15)	0.229070 (15)	0.02035 (11)	
K	0.24658 (3)	0.14983 (3)	0.04404 (3)	0.03328 (16)	
S	0.00525 (5)	0.04132 (4)	0.31693 (3)	0.0438 (2)	
O1	0.35902 (15)	−0.05101 (13)	0.44571 (15)	0.0879 (10)	
O2	−0.00865 (15)	0.30157 (11)	0.44698 (11)	0.0663 (7)	
O3	−0.38816 (13)	0.07683 (15)	0.18152 (12)	0.0819 (9)	
O4	−0.10604 (18)	−0.25034 (13)	0.24074 (15)	0.0954 (11)	
O5	0.17791 (11)	0.21220 (9)	−0.06246 (9)	0.0422 (5)	
O6	0.30993 (11)	0.14053 (10)	−0.05905 (9)	0.0468 (5)	
O7	0.23261 (11)	0.01974 (9)	0.05618 (8)	0.0381 (5)	
O8	0.11064 (11)	0.10019 (10)	0.06719 (10)	0.0475 (5)	
O9	0.26318 (11)	0.25534 (9)	0.12075 (8)	0.0413 (5)	
O10	0.38436 (10)	0.17024 (8)	0.12576 (9)	0.0359 (5)	
N1	0.12768 (11)	0.00670 (9)	0.22448 (9)	0.0227 (5)	
N2	0.12981 (11)	0.13726 (9)	0.25363 (9)	0.0249 (5)	
N3	−0.02512 (12)	0.14767 (9)	0.19543 (10)	0.0269 (5)	
N4	−0.02802 (11)	0.01795 (9)	0.16376 (9)	0.0236 (5)	
N5	0.29610 (13)	0.02326 (11)	0.38752 (10)	0.0374 (6)	
HN5	0.2535	0.0446	0.3810	0.045*	
N6	0.01197 (18)	0.25758 (12)	0.36235 (12)	0.0554 (8)	
HN6	0.0154	0.2213	0.3453	0.067*	
N7	−0.26586 (13)	0.08270 (12)	0.16940 (11)	0.0416 (6)	
HN7	−0.2193	0.0753	0.1882	0.050*	
N8	−0.02707 (13)	−0.17059 (10)	0.23845 (10)	0.0357 (6)	
HN8	−0.0134	−0.1341	0.2543	0.043*	
N9	0.11215 (14)	0.23331 (12)	0.04717 (11)	0.0453 (6)	
N10	0.38315 (12)	0.06590 (10)	0.04173 (10)	0.0341 (5)	
C1	0.11518 (14)	−0.05461 (11)	0.20873 (11)	0.0230 (6)	
C2	0.18483 (15)	−0.08910 (12)	0.22589 (11)	0.0290 (6)	
H2	0.1913	−0.1324	0.2208	0.035*	
C3	0.23912 (15)	−0.04840 (12)	0.25046 (12)	0.0292 (6)	
H3	0.2910	−0.0575	0.2654	0.035*	
C4	0.20305 (14)	0.01169 (11)	0.24964 (11)	0.0241 (6)	
C5	0.23965 (14)	0.06684 (12)	0.27127 (11)	0.0250 (6)	
C6	0.20471 (14)	0.12472 (12)	0.27393 (11)	0.0260 (6)	
C7	0.24232 (15)	0.17981 (12)	0.30131 (12)	0.0320 (6)	
H7	0.2946	0.1836	0.3184	0.038*	
C8	0.18931 (15)	0.22458 (12)	0.29799 (12)	0.0331 (7)	
H8	0.1969	0.2657	0.3129	0.040*	
C9	0.11896 (14)	0.19838 (11)	0.26752 (11)	0.0264 (6)	
C10	0.05056 (15)	0.23077 (12)	0.25186 (12)	0.0281 (6)	
C11	−0.01628 (15)	0.20707 (12)	0.21737 (12)	0.0303 (6)	
C12	−0.08348 (16)	0.24277 (13)	0.19642 (15)	0.0433 (8)	
H12	−0.0921	0.2847	0.2062	0.052*	
C13	−0.13204 (16)	0.20557 (13)	0.16026 (15)	0.0449 (8)	
H13	−0.1809	0.2166	0.1391	0.054*	
C14	−0.09595 (14)	0.14579 (12)	0.15975 (12)	0.0304 (6)	
C15	−0.12845 (14)	0.09360 (12)	0.12770 (12)	0.0283 (6)	
C16	−0.09700 (13)	0.03408 (11)	0.13037 (11)	0.0246 (6)	
C17	−0.13244 (15)	−0.01987 (12)	0.09970 (12)	0.0298 (6)	
H17	−0.1802	−0.0212	0.0735	0.036*	
C18	−0.08482 (15)	−0.06829 (12)	0.11526 (12)	0.0292 (6)	
H18	−0.0932	−0.1101	0.1025	0.035*	
C19	−0.01910 (14)	−0.04436 (11)	0.15478 (11)	0.0240 (6)	
C20	0.04683 (14)	−0.07882 (11)	0.17727 (11)	0.0237 (6)	
C21	0.32344 (14)	0.06323 (11)	0.29518 (12)	0.0260 (6)	
C22	0.37551 (15)	0.08190 (12)	0.26052 (13)	0.0308 (6)	
H22	0.3578	0.0977	0.2215	0.037*	
C23	0.45264 (15)	0.07790 (12)	0.28170 (14)	0.0357 (7)	
H23	0.4876	0.0905	0.2574	0.043*	
C24	0.47844 (15)	0.05530 (13)	0.33885 (14)	0.0378 (7)	
H24	0.5314	0.0524	0.3537	0.045*	
C25	0.42799 (15)	0.03695 (13)	0.37424 (13)	0.0360 (7)	
H25	0.4463	0.0219	0.4135	0.043*	
C26	0.35034 (15)	0.04034 (12)	0.35277 (12)	0.0308 (6)	
C27	0.30111 (17)	−0.02178 (14)	0.42973 (14)	0.0442 (8)	
C28	0.2289 (2)	−0.03436 (15)	0.45455 (15)	0.0511 (8)	
C29	0.2517 (3)	−0.0560 (2)	0.5186 (2)	0.0941 (13)	
H29A	0.2896	−0.0892	0.5207	0.141*	
H29B	0.2070	−0.0718	0.5329	0.141*	
H29C	0.2734	−0.0211	0.5437	0.141*	
C30	0.1771 (2)	0.02138 (19)	0.4526 (2)	0.0719 (11)	
H30A	0.2057	0.0566	0.4726	0.108*	
H30B	0.1350	0.0113	0.4729	0.108*	
H30C	0.1570	0.0323	0.4109	0.108*	
C31	0.1864 (3)	−0.0861 (2)	0.4166 (2)	0.0855 (11)	
H31A	0.1708	−0.0716	0.3754	0.128*	
H31B	0.1414	−0.0977	0.4328	0.128*	
H31C	0.2197	−0.1223	0.4172	0.128*	
C32	0.04810 (15)	0.29677 (12)	0.27287 (13)	0.0324 (7)	
C33	0.06150 (16)	0.34574 (13)	0.23722 (15)	0.0394 (7)	
H33	0.0751	0.3375	0.1996	0.047*	
C34	0.05545 (17)	0.40705 (14)	0.25541 (16)	0.0460 (8)	
H34	0.0651	0.4404	0.2306	0.055*	
C35	0.03542 (19)	0.41843 (14)	0.30961 (17)	0.0515 (9)	
H35	0.0303	0.4601	0.3219	0.062*	
C36	0.02253 (19)	0.37037 (15)	0.34681 (16)	0.0522 (9)	
H36	0.0099	0.3791	0.3847	0.063*	
C37	0.02810 (17)	0.30929 (13)	0.32848 (14)	0.0405 (7)	
C38	−0.00786 (16)	0.25520 (15)	0.41683 (13)	0.0410 (8)	
C39	−0.02581 (18)	0.19145 (15)	0.43781 (14)	0.0454 (8)	
C40	−0.0543 (2)	0.1989 (2)	0.49710 (16)	0.0730 (11)	
H40A	−0.0147	0.2184	0.5267	0.110*	
H40B	−0.0666	0.1580	0.5116	0.110*	
H40C	−0.0998	0.2251	0.4908	0.110*	
C41	0.0468 (2)	0.15148 (17)	0.44790 (18)	0.0649 (10)	
H41A	0.0641	0.1459	0.4099	0.097*	
H41B	0.0359	0.1108	0.4637	0.097*	
H41C	0.0864	0.1723	0.4765	0.097*	
C42	−0.08738 (19)	0.15999 (16)	0.39233 (16)	0.0546 (9)	
H42A	−0.1321	0.1870	0.3839	0.082*	
H42B	−0.1014	0.1204	0.4086	0.082*	
H42C	−0.0683	0.1524	0.3553	0.082*	
C43	−0.20380 (15)	0.10628 (12)	0.08782 (13)	0.0328 (7)	
C44	−0.20574 (18)	0.12848 (14)	0.03058 (13)	0.0426 (7)	
H44	−0.1603	0.1301	0.0149	0.051*	
C45	−0.2733 (2)	0.14838 (14)	−0.00416 (15)	0.0527 (9)	
H45	−0.2739	0.1640	−0.0433	0.063*	
C46	−0.3394 (2)	0.14545 (15)	0.01823 (17)	0.0555 (10)	
H46	−0.3856	0.1592	−0.0055	0.067*	
C47	−0.33912 (17)	0.12266 (15)	0.07501 (15)	0.0479 (8)	
H47	−0.3851	0.1205	0.0900	0.057*	
C48	−0.27131 (15)	0.10290 (13)	0.11028 (13)	0.0364 (7)	
C49	−0.32059 (17)	0.07264 (15)	0.20256 (16)	0.0484 (8)	
C50	−0.29353 (17)	0.05719 (15)	0.26818 (15)	0.0465 (8)	
C51	−0.20843 (18)	0.0463 (2)	0.28585 (17)	0.0646 (10)	
H51A	−0.1931	0.0131	0.2611	0.097*	
H51B	−0.1962	0.0341	0.3280	0.097*	
H51C	−0.1812	0.0845	0.2798	0.097*	
C52	−0.3360 (2)	−0.00087 (18)	0.2830 (2)	0.0731 (11)	
H52A	−0.3908	0.0066	0.2731	0.110*	
H52B	−0.3214	−0.0102	0.3257	0.110*	
H52C	−0.3229	−0.0361	0.2596	0.110*	
C53	−0.3165 (2)	0.11162 (18)	0.30420 (17)	0.0674 (10)	
H53A	−0.2882	0.1488	0.2967	0.101*	
H53B	−0.3051	0.1015	0.3469	0.101*	
H53C	−0.3710	0.1194	0.2921	0.101*	
C54	0.04742 (14)	−0.14673 (11)	0.16275 (12)	0.0264 (6)	
C55	0.08766 (15)	−0.16758 (12)	0.12005 (12)	0.0311 (6)	
H55	0.1117	−0.1380	0.0988	0.037*	
C56	0.09408 (16)	−0.22982 (13)	0.10731 (13)	0.0362 (7)	
H56	0.1218	−0.2428	0.0777	0.043*	
C57	0.05944 (15)	−0.27283 (12)	0.13851 (13)	0.0360 (7)	
H57	0.0642	−0.3159	0.1308	0.043*	
C58	0.01841 (16)	−0.25414 (12)	0.18040 (13)	0.0342 (7)	
H58	−0.0057	−0.2842	0.2010	0.041*	
C59	0.01175 (14)	−0.19128 (12)	0.19310 (12)	0.0280 (6)	
C60	−0.08233 (19)	−0.20026 (15)	0.26013 (15)	0.0465 (8)	
C61	−0.1158 (2)	−0.16870 (16)	0.30932 (16)	0.0539 (9)	
C62	−0.1268 (3)	−0.2177 (2)	0.3550 (2)	0.0813 (9)	0.84
H62A	−0.0777	−0.2361	0.3722	0.122*	0.84
H62B	−0.1610	−0.2501	0.3354	0.122*	0.84
H62C	−0.1490	−0.1984	0.3868	0.122*	0.84
C63	−0.0624 (3)	−0.1185 (3)	0.3436 (2)	0.0813 (9)	0.84
H63A	−0.0826	−0.1048	0.3786	0.122*	0.84
H63B	−0.0593	−0.0830	0.3172	0.122*	0.84
H63C	−0.0117	−0.1362	0.3566	0.122*	0.84
C64	−0.1933 (3)	−0.1425 (3)	0.2813 (2)	0.0813 (9)	0.84
H64A	−0.2248	−0.1757	0.2602	0.122*	0.84
H64B	−0.1868	−0.1096	0.2529	0.122*	0.84
H64C	−0.2180	−0.1254	0.3127	0.122*	0.84
C62A	−0.0701 (13)	−0.1780 (13)	0.3665 (7)	0.0813 (9)	0.16
H62D	−0.0323	−0.1447	0.3746	0.122*	0.16
H62E	−0.0444	−0.2182	0.3673	0.122*	0.16
H62F	−0.1022	−0.1774	0.3970	0.122*	0.16
C63A	−0.1457 (16)	−0.1036 (9)	0.2853 (11)	0.0813 (9)	0.16
H63D	−0.1841	−0.0891	0.3077	0.122*	0.16
H63E	−0.1684	−0.1069	0.2429	0.122*	0.16
H63F	−0.1035	−0.0740	0.2904	0.122*	0.16
C64A	−0.1985 (10)	−0.2025 (12)	0.2985 (11)	0.0813 (9)	0.16
H64D	−0.1918	−0.2476	0.2987	0.122*	0.16
H64E	−0.2280	−0.1894	0.2599	0.122*	0.16
H64F	−0.2256	−0.1906	0.3305	0.122*	0.16
C65	0.08101 (17)	0.25233 (16)	−0.01462 (15)	0.0504 (8)	
H65A	0.0482	0.2891	−0.0136	0.061*	
H65B	0.0489	0.2184	−0.0348	0.061*	
C66	0.14054 (18)	0.26764 (15)	−0.05033 (15)	0.0495 (8)	
H66A	0.1170	0.2878	−0.0883	0.059*	
H66B	0.1777	0.2969	−0.0278	0.059*	
C67	0.23079 (19)	0.22303 (17)	−0.10065 (15)	0.0551 (9)	
H67A	0.2700	0.2527	−0.0815	0.066*	
H67B	0.2044	0.2414	−0.1387	0.066*	
C68	0.26767 (19)	0.16304 (17)	−0.11297 (14)	0.0529 (9)	
H68A	0.2285	0.1324	−0.1300	0.063*	
H68B	0.3014	0.1699	−0.1422	0.063*	
C69	0.35155 (18)	0.08645 (16)	−0.06728 (14)	0.0471 (8)	
H69A	0.3747	0.0910	−0.1033	0.057*	
H69B	0.3173	0.0500	−0.0730	0.057*	
C70	0.41275 (17)	0.07682 (15)	−0.01301 (14)	0.0436 (8)	
H70A	0.4443	0.0409	−0.0202	0.052*	
H70B	0.4459	0.1139	−0.0074	0.052*	
C71	0.35853 (17)	0.00097 (13)	0.04389 (13)	0.0387 (7)	
H71A	0.4034	−0.0254	0.0582	0.046*	
H71B	0.3360	−0.0128	0.0030	0.046*	
C72	0.30177 (17)	−0.00817 (13)	0.08382 (13)	0.0401 (7)	
H72A	0.2940	−0.0531	0.0900	0.048*	
H72B	0.3203	0.0112	0.1232	0.048*	
C73	0.17263 (18)	0.00352 (15)	0.08625 (15)	0.0482 (8)	
H73A	0.1859	0.0159	0.1288	0.058*	
H73B	0.1649	−0.0420	0.0846	0.058*	
C74	0.10171 (18)	0.03529 (16)	0.05767 (15)	0.0501 (9)	
H74A	0.0910	0.0263	0.0143	0.060*	
H74B	0.0585	0.0200	0.0752	0.060*	
C75	0.04258 (18)	0.13371 (17)	0.04986 (17)	0.0559 (9)	
H75A	0.0013	0.1137	0.0665	0.067*	
H75B	0.0280	0.1341	0.0058	0.067*	
C76	0.05434 (19)	0.19858 (18)	0.07278 (18)	0.0610 (10)	
H76A	0.0055	0.2212	0.0635	0.073*	
H76B	0.0700	0.1973	0.1168	0.073*	
C77	0.13663 (19)	0.28846 (16)	0.08408 (16)	0.0559 (9)	
H77A	0.0917	0.3073	0.0967	0.067*	
H77B	0.1577	0.3194	0.0594	0.067*	
C78	0.19482 (19)	0.27468 (17)	0.13848 (15)	0.0542 (9)	
H78A	0.2044	0.3123	0.1638	0.065*	
H78B	0.1761	0.2415	0.1621	0.065*	
C79	0.32435 (17)	0.25103 (15)	0.16984 (13)	0.0436 (8)	
H79A	0.3122	0.2203	0.1990	0.052*	
H79B	0.3327	0.2918	0.1901	0.052*	
C80	0.39374 (16)	0.23183 (14)	0.14824 (14)	0.0401 (7)	
H80A	0.4032	0.2603	0.1163	0.048*	
H80B	0.4379	0.2338	0.1814	0.048*	
C81	0.45188 (16)	0.14729 (13)	0.10798 (15)	0.0409 (7)	
H81A	0.4961	0.1539	0.1404	0.049*	
H81B	0.4610	0.1697	0.0720	0.049*	
C82	0.44180 (16)	0.07922 (13)	0.09470 (14)	0.0405 (7)	
H82A	0.4907	0.0617	0.0883	0.049*	
H82B	0.4280	0.0581	0.1298	0.049*	

### Atomic displacement parameters (Å^2^)

**Table:**

	*U*^11^	*U*^22^	*U*^33^	*U*^12^	*U*^13^	*U*^23^
Fe	0.01704 (19)	0.0154 (2)	0.0283 (2)	−0.00017 (14)	0.00341 (15)	−0.00347 (14)
K	0.0293 (3)	0.0334 (4)	0.0363 (3)	−0.0034 (3)	0.0036 (3)	0.0028 (3)
S	0.0485 (5)	0.0474 (5)	0.0400 (4)	−0.0011 (4)	0.0204 (4)	−0.0003 (3)
O1	0.0568 (17)	0.0754 (19)	0.135 (3)	0.0238 (15)	0.0276 (17)	0.0608 (19)
O2	0.0808 (18)	0.0550 (15)	0.0675 (16)	−0.0020 (13)	0.0252 (14)	−0.0329 (13)
O3	0.0221 (13)	0.138 (3)	0.0820 (19)	0.0016 (14)	0.0007 (12)	0.0137 (17)
O4	0.112 (2)	0.0556 (18)	0.141 (3)	−0.0473 (17)	0.083 (2)	−0.0369 (18)
O5	0.0377 (12)	0.0403 (12)	0.0470 (12)	−0.0046 (10)	0.0035 (10)	0.0119 (9)
O6	0.0394 (12)	0.0632 (15)	0.0381 (12)	0.0054 (11)	0.0077 (10)	0.0104 (10)
O7	0.0401 (12)	0.0375 (11)	0.0389 (11)	−0.0100 (9)	0.0130 (9)	0.0057 (9)
O8	0.0331 (12)	0.0490 (14)	0.0616 (14)	−0.0078 (10)	0.0120 (10)	0.0029 (11)
O9	0.0397 (12)	0.0452 (12)	0.0368 (11)	0.0075 (10)	0.0014 (9)	−0.0046 (9)
O10	0.0298 (10)	0.0298 (11)	0.0463 (12)	−0.0031 (8)	0.0023 (9)	−0.0008 (9)
N1	0.0207 (11)	0.0191 (12)	0.0283 (11)	−0.0018 (9)	0.0046 (9)	−0.0009 (9)
N2	0.0230 (12)	0.0188 (12)	0.0320 (12)	0.0005 (9)	0.0027 (9)	−0.0023 (9)
N3	0.0236 (12)	0.0189 (12)	0.0372 (13)	−0.0006 (9)	0.0027 (10)	−0.0044 (9)
N4	0.0220 (11)	0.0183 (11)	0.0307 (12)	0.0012 (9)	0.0054 (9)	−0.0031 (9)
N5	0.0307 (13)	0.0445 (15)	0.0372 (13)	0.0070 (11)	0.0072 (11)	0.0071 (11)
N6	0.090 (2)	0.0294 (15)	0.0535 (17)	−0.0027 (14)	0.0303 (16)	−0.0174 (12)
N7	0.0185 (12)	0.0541 (16)	0.0495 (16)	0.0050 (11)	−0.0011 (11)	−0.0054 (12)
N8	0.0442 (14)	0.0206 (12)	0.0445 (14)	−0.0033 (11)	0.0137 (12)	−0.0002 (10)
N9	0.0373 (14)	0.0464 (16)	0.0503 (16)	0.0062 (12)	0.0031 (12)	0.0006 (12)
N10	0.0276 (12)	0.0309 (13)	0.0435 (14)	−0.0035 (10)	0.0060 (11)	0.0038 (10)
C1	0.0226 (14)	0.0196 (14)	0.0278 (14)	−0.0014 (11)	0.0074 (11)	0.0004 (11)
C2	0.0312 (15)	0.0203 (14)	0.0352 (15)	0.0030 (12)	0.0055 (12)	0.0017 (11)
C3	0.0236 (14)	0.0259 (15)	0.0365 (15)	0.0027 (12)	0.0012 (12)	0.0002 (12)
C4	0.0233 (14)	0.0224 (14)	0.0266 (14)	0.0007 (11)	0.0044 (11)	0.0010 (11)
C5	0.0223 (13)	0.0265 (15)	0.0260 (14)	−0.0017 (11)	0.0040 (11)	−0.0004 (11)
C6	0.0234 (14)	0.0234 (14)	0.0304 (14)	−0.0010 (11)	0.0031 (11)	0.0007 (11)
C7	0.0264 (15)	0.0259 (15)	0.0408 (16)	−0.0037 (12)	−0.0012 (12)	−0.0038 (12)
C8	0.0327 (16)	0.0224 (15)	0.0422 (17)	−0.0057 (13)	0.0020 (13)	−0.0089 (12)
C9	0.0280 (15)	0.0216 (14)	0.0295 (14)	−0.0009 (11)	0.0056 (11)	−0.0024 (11)
C10	0.0299 (15)	0.0191 (14)	0.0361 (15)	0.0004 (12)	0.0085 (12)	−0.0058 (11)
C11	0.0278 (15)	0.0217 (15)	0.0405 (16)	0.0005 (12)	0.0042 (12)	−0.0066 (12)
C12	0.0349 (17)	0.0218 (15)	0.069 (2)	0.0075 (13)	−0.0017 (15)	−0.0131 (14)
C13	0.0281 (16)	0.0302 (17)	0.070 (2)	0.0088 (13)	−0.0089 (15)	−0.0117 (15)
C14	0.0229 (14)	0.0239 (15)	0.0426 (16)	0.0026 (12)	0.0015 (12)	−0.0045 (12)
C15	0.0224 (14)	0.0272 (15)	0.0347 (15)	0.0001 (12)	0.0035 (12)	−0.0031 (12)
C16	0.0197 (13)	0.0236 (15)	0.0310 (14)	−0.0022 (11)	0.0064 (11)	−0.0020 (11)
C17	0.0229 (14)	0.0282 (15)	0.0364 (15)	−0.0027 (12)	0.0005 (12)	−0.0070 (12)
C18	0.0285 (15)	0.0206 (14)	0.0376 (15)	−0.0036 (12)	0.0040 (12)	−0.0069 (12)
C19	0.0237 (14)	0.0206 (14)	0.0286 (14)	−0.0035 (11)	0.0073 (11)	−0.0003 (11)
C20	0.0246 (14)	0.0179 (13)	0.0292 (14)	−0.0014 (11)	0.0065 (11)	−0.0005 (11)
C21	0.0231 (14)	0.0167 (13)	0.0373 (15)	0.0009 (11)	0.0034 (12)	−0.0031 (11)
C22	0.0279 (15)	0.0236 (15)	0.0402 (16)	−0.0021 (12)	0.0047 (12)	−0.0021 (12)
C23	0.0265 (16)	0.0268 (16)	0.0554 (19)	−0.0025 (12)	0.0112 (14)	−0.0032 (13)
C24	0.0191 (14)	0.0331 (16)	0.058 (2)	−0.0006 (12)	−0.0002 (14)	−0.0050 (14)
C25	0.0306 (16)	0.0340 (16)	0.0399 (16)	0.0034 (13)	−0.0028 (13)	0.0003 (13)
C26	0.0295 (15)	0.0260 (15)	0.0366 (16)	0.0014 (12)	0.0051 (13)	−0.0021 (12)
C27	0.0391 (18)	0.0358 (17)	0.058 (2)	0.0070 (15)	0.0095 (15)	0.0110 (15)
C28	0.058 (2)	0.045 (2)	0.054 (2)	0.0021 (17)	0.0192 (17)	0.0142 (16)
C29	0.129 (4)	0.070	0.092	0.027 (3)	0.044 (3)	0.037 (2)
C30	0.065 (2)	0.070 (3)	0.091 (3)	0.011 (2)	0.041 (2)	0.022 (2)
C31	0.103 (3)	0.070	0.092	−0.032 (2)	0.039 (3)	−0.017 (2)
C32	0.0243 (14)	0.0227 (15)	0.0475 (17)	0.0019 (12)	−0.0004 (12)	−0.0086 (13)
C33	0.0349 (16)	0.0241 (16)	0.058 (2)	−0.0007 (13)	0.0056 (14)	−0.0039 (14)
C34	0.0384 (18)	0.0236 (16)	0.072 (2)	−0.0032 (13)	−0.0015 (16)	−0.0044 (15)
C35	0.048 (2)	0.0244 (17)	0.078 (3)	0.0039 (14)	0.0018 (18)	−0.0190 (17)
C36	0.060 (2)	0.0347 (19)	0.063 (2)	0.0000 (16)	0.0127 (17)	−0.0220 (17)
C37	0.0410 (17)	0.0284 (17)	0.0508 (19)	−0.0020 (13)	0.0055 (14)	−0.0133 (14)
C38	0.0303 (16)	0.047 (2)	0.0444 (18)	0.0055 (14)	0.0047 (14)	−0.0183 (15)
C39	0.0434 (18)	0.051 (2)	0.0429 (18)	−0.0002 (15)	0.0113 (14)	−0.0109 (15)
C40	0.089 (3)	0.084 (3)	0.053 (2)	−0.005 (2)	0.028 (2)	−0.012 (2)
C41	0.059 (2)	0.059 (2)	0.072 (3)	0.0127 (19)	0.0016 (19)	0.0052 (19)
C42	0.050 (2)	0.055 (2)	0.062 (2)	−0.0098 (17)	0.0176 (17)	−0.0183 (17)
C43	0.0290 (15)	0.0209 (15)	0.0449 (17)	0.0026 (12)	−0.0028 (13)	−0.0075 (12)
C44	0.0448 (19)	0.0342 (17)	0.0443 (18)	0.0010 (14)	−0.0036 (15)	−0.0031 (14)
C45	0.063 (2)	0.0356 (18)	0.050 (2)	0.0066 (17)	−0.0154 (18)	0.0010 (15)
C46	0.049 (2)	0.0381 (19)	0.066 (2)	0.0114 (16)	−0.0240 (18)	−0.0083 (17)
C47	0.0306 (17)	0.0455 (19)	0.062 (2)	0.0097 (14)	−0.0064 (15)	−0.0137 (16)
C48	0.0276 (16)	0.0302 (16)	0.0472 (18)	0.0038 (12)	−0.0039 (13)	−0.0122 (13)
C49	0.0242 (18)	0.051 (2)	0.068 (2)	−0.0001 (14)	0.0042 (16)	−0.0089 (17)
C50	0.0284 (16)	0.052 (2)	0.060 (2)	0.0016 (14)	0.0097 (15)	0.0012 (16)
C51	0.0372 (19)	0.097 (3)	0.058 (2)	0.0068 (19)	0.0047 (16)	0.012 (2)
C52	0.052 (2)	0.059 (2)	0.110 (3)	−0.0007 (19)	0.020 (2)	0.009 (2)
C53	0.075 (3)	0.063 (2)	0.066 (2)	0.009 (2)	0.018 (2)	−0.0035 (19)
C54	0.0214 (13)	0.0196 (14)	0.0354 (15)	0.0000 (11)	−0.0018 (12)	−0.0002 (11)
C55	0.0271 (15)	0.0257 (15)	0.0402 (16)	−0.0027 (12)	0.0053 (12)	−0.0039 (12)
C56	0.0332 (16)	0.0272 (16)	0.0478 (18)	0.0042 (13)	0.0062 (13)	−0.0094 (13)
C57	0.0332 (16)	0.0178 (14)	0.0523 (18)	0.0050 (12)	−0.0040 (14)	−0.0042 (13)
C58	0.0360 (16)	0.0190 (15)	0.0440 (17)	−0.0003 (12)	−0.0019 (13)	0.0046 (12)
C59	0.0250 (14)	0.0230 (15)	0.0333 (15)	0.0003 (11)	−0.0014 (12)	0.0006 (11)
C60	0.054 (2)	0.0323 (19)	0.058 (2)	−0.0016 (16)	0.0210 (17)	0.0100 (15)
C61	0.064 (2)	0.045 (2)	0.060 (2)	0.0131 (17)	0.0294 (18)	0.0136 (16)
C62	0.104 (2)	0.081 (2)	0.0676 (18)	0.0104 (18)	0.0387 (17)	0.0036 (16)
C63	0.104 (2)	0.081 (2)	0.0676 (18)	0.0104 (18)	0.0387 (17)	0.0036 (16)
C64	0.104 (2)	0.081 (2)	0.0676 (18)	0.0104 (18)	0.0387 (17)	0.0036 (16)
C62A	0.104 (2)	0.081 (2)	0.0676 (18)	0.0104 (18)	0.0387 (17)	0.0036 (16)
C63A	0.104 (2)	0.081 (2)	0.0676 (18)	0.0104 (18)	0.0387 (17)	0.0036 (16)
C64A	0.104 (2)	0.081 (2)	0.0676 (18)	0.0104 (18)	0.0387 (17)	0.0036 (16)
C65	0.0354 (18)	0.051 (2)	0.059 (2)	0.0097 (15)	−0.0065 (15)	0.0035 (16)
C66	0.0473 (19)	0.0405 (19)	0.053 (2)	−0.0019 (16)	−0.0111 (16)	0.0119 (15)
C67	0.047 (2)	0.062 (2)	0.054 (2)	−0.0092 (18)	0.0060 (16)	0.0286 (17)
C68	0.0452 (19)	0.076 (3)	0.0386 (18)	−0.0037 (18)	0.0109 (15)	0.0184 (17)
C69	0.0448 (19)	0.056 (2)	0.0444 (18)	−0.0059 (16)	0.0184 (15)	0.0014 (15)
C70	0.0325 (17)	0.0466 (19)	0.0537 (19)	−0.0012 (14)	0.0133 (14)	0.0065 (15)
C71	0.0441 (18)	0.0269 (15)	0.0442 (17)	−0.0032 (13)	0.0058 (14)	−0.0008 (13)
C72	0.0511 (19)	0.0268 (16)	0.0412 (17)	−0.0066 (14)	0.0050 (15)	0.0031 (13)
C73	0.056 (2)	0.0424 (19)	0.0526 (19)	−0.0136 (16)	0.0281 (17)	0.0075 (15)
C74	0.0439 (19)	0.059 (2)	0.052 (2)	−0.0235 (17)	0.0217 (16)	−0.0028 (16)
C75	0.0327 (18)	0.066 (2)	0.070 (2)	−0.0063 (17)	0.0125 (16)	0.0089 (19)
C76	0.0387 (19)	0.075 (3)	0.073 (2)	0.0097 (18)	0.0201 (18)	0.004 (2)
C77	0.047 (2)	0.054 (2)	0.063 (2)	0.0179 (17)	0.0031 (17)	−0.0108 (17)
C78	0.050 (2)	0.062 (2)	0.050 (2)	0.0131 (17)	0.0098 (16)	−0.0108 (17)
C79	0.0476 (19)	0.0465 (19)	0.0340 (16)	0.0014 (15)	0.0003 (14)	−0.0049 (14)
C80	0.0374 (17)	0.0359 (17)	0.0440 (17)	−0.0052 (14)	0.0000 (14)	−0.0067 (13)
C81	0.0258 (15)	0.0361 (17)	0.058 (2)	0.0001 (13)	−0.0002 (14)	0.0008 (14)
C82	0.0293 (16)	0.0355 (17)	0.0530 (19)	0.0041 (13)	−0.0024 (14)	0.0035 (14)

### Geometric parameters (Å, °)

**Table:**

Fe—N1	2.087 (2)	C36—C37	1.389 (4)
Fe—N3	2.099 (2)	C36—H36	0.9500
Fe—N4	2.104 (2)	C38—C39	1.508 (5)
Fe—N2	2.115 (2)	C39—C42	1.526 (4)
Fe—S	2.3123 (8)	C39—C40	1.537 (5)
K—O6	2.797 (2)	C39—C41	1.542 (5)
K—O8	2.799 (2)	C40—H40A	0.9800
K—O7	2.831 (2)	C40—H40B	0.9800
K—O10	2.8402 (19)	C40—H40C	0.9800
K—O5	2.846 (2)	C41—H41A	0.9800
K—O9	2.848 (2)	C41—H41B	0.9800
K—N9	3.018 (3)	C41—H41C	0.9800
K—N10	3.052 (2)	C42—H42A	0.9800
O1—C27	1.212 (4)	C42—H42B	0.9800
O2—C38	1.213 (4)	C42—H42C	0.9800
O3—C49	1.221 (4)	C43—C44	1.383 (4)
O4—C60	1.212 (4)	C43—C48	1.400 (4)
O5—C67	1.419 (4)	C44—C45	1.387 (4)
O5—C66	1.421 (4)	C44—H44	0.9500
O6—C68	1.405 (4)	C45—C46	1.375 (5)
O6—C69	1.414 (4)	C45—H45	0.9500
O7—C72	1.417 (3)	C46—C47	1.382 (5)
O7—C73	1.421 (3)	C46—H46	0.9500
O8—C75	1.410 (4)	C47—C48	1.395 (4)
O8—C74	1.419 (4)	C47—H47	0.9500
O9—C79	1.418 (3)	C49—C50	1.520 (5)
O9—C78	1.422 (4)	C50—C51	1.523 (4)
O10—C80	1.420 (3)	C50—C53	1.530 (5)
O10—C81	1.433 (3)	C50—C52	1.533 (5)
N1—C4	1.372 (3)	C51—H51A	0.9800
N1—C1	1.376 (3)	C51—H51B	0.9800
N2—C6	1.365 (3)	C51—H51C	0.9800
N2—C9	1.376 (3)	C52—H52A	0.9800
N3—C11	1.372 (3)	C52—H52B	0.9800
N3—C14	1.376 (3)	C52—H52C	0.9800
N4—C19	1.371 (3)	C53—H53A	0.9800
N4—C16	1.372 (3)	C53—H53B	0.9800
N5—C27	1.357 (4)	C53—H53C	0.9800
N5—C26	1.411 (3)	C54—C55	1.388 (4)
N5—HN5	0.8800	C54—C59	1.403 (4)
N6—C38	1.353 (4)	C55—C56	1.381 (4)
N6—C37	1.414 (4)	C55—H55	0.9500
N6—HN6	0.8800	C56—C57	1.382 (4)
N7—C49	1.361 (4)	C56—H56	0.9500
N7—C48	1.401 (4)	C57—C58	1.368 (4)
N7—HN7	0.8800	C57—H57	0.9500
N8—C60	1.347 (4)	C58—C59	1.394 (4)
N8—C59	1.419 (3)	C58—H58	0.9500
N8—HN8	0.8800	C60—C61	1.524 (5)
N9—C65	1.473 (4)	C61—C62A	1.418 (15)
N9—C77	1.474 (4)	C61—C62	1.520 (6)
N9—C76	1.482 (4)	C61—C64	1.527 (6)
N10—C70	1.461 (4)	C61—C63	1.555 (6)
N10—C71	1.470 (4)	C61—C63A	1.563 (15)
N10—C82	1.475 (4)	C61—C64A	1.630 (16)
C1—C20	1.402 (4)	C62—H62A	0.9800
C1—C2	1.444 (4)	C62—H62B	0.9800
C2—C3	1.352 (4)	C62—H62C	0.9800
C2—H2	0.9500	C63—H63A	0.9800
C3—C4	1.445 (4)	C63—H63B	0.9800
C3—H3	0.9500	C63—H63C	0.9800
C4—C5	1.402 (4)	C64—H64A	0.9800
C5—C6	1.401 (4)	C64—H64B	0.9800
C5—C21	1.503 (4)	C64—H64C	0.9800
C6—C7	1.448 (4)	C62A—H62D	0.9800
C7—C8	1.346 (4)	C62A—H62E	0.9800
C7—H7	0.9500	C62A—H62F	0.9800
C8—C9	1.438 (4)	C63A—H63D	0.9800
C8—H8	0.9500	C63A—H63E	0.9800
C9—C10	1.399 (4)	C63A—H63F	0.9800
C10—C11	1.403 (4)	C64A—H64D	0.9800
C10—C32	1.503 (4)	C64A—H64E	0.9800
C11—C12	1.435 (4)	C64A—H64F	0.9800
C12—C13	1.347 (4)	C65—C66	1.493 (5)
C12—H12	0.9500	C65—H65A	0.9900
C13—C14	1.442 (4)	C65—H65B	0.9900
C13—H13	0.9500	C66—H66A	0.9900
C14—C15	1.407 (4)	C66—H66B	0.9900
C15—C16	1.397 (4)	C67—C68	1.501 (5)
C15—C43	1.508 (4)	C67—H67A	0.9900
C16—C17	1.441 (4)	C67—H67B	0.9900
C17—C18	1.353 (4)	C68—H68A	0.9900
C17—H17	0.9500	C68—H68B	0.9900
C18—C19	1.440 (4)	C69—C70	1.508 (4)
C18—H18	0.9500	C69—H69A	0.9900
C19—C20	1.410 (4)	C69—H69B	0.9900
C20—C54	1.500 (3)	C70—H70A	0.9900
C21—C22	1.388 (4)	C70—H70B	0.9900
C21—C26	1.401 (4)	C71—C72	1.497 (4)
C22—C23	1.381 (4)	C71—H71A	0.9900
C22—H22	0.9500	C71—H71B	0.9900
C23—C24	1.387 (4)	C72—H72A	0.9900
C23—H23	0.9500	C72—H72B	0.9900
C24—C25	1.375 (4)	C73—C74	1.485 (5)
C24—H24	0.9500	C73—H73A	0.9900
C25—C26	1.390 (4)	C73—H73B	0.9900
C25—H25	0.9500	C74—H74A	0.9900
C27—C28	1.530 (4)	C74—H74B	0.9900
C28—C30	1.514 (5)	C75—C76	1.493 (5)
C28—C29	1.514 (5)	C75—H75A	0.9900
C28—C31	1.525 (5)	C75—H75B	0.9900
C29—H29A	0.9800	C76—H76A	0.9900
C29—H29B	0.9800	C76—H76B	0.9900
C29—H29C	0.9800	C77—C78	1.495 (5)
C30—H30A	0.9800	C77—H77A	0.9900
C30—H30B	0.9800	C77—H77B	0.9900
C30—H30C	0.9800	C78—H78A	0.9900
C31—H31A	0.9800	C78—H78B	0.9900
C31—H31B	0.9800	C79—C80	1.479 (4)
C31—H31C	0.9800	C79—H79A	0.9900
C32—C33	1.379 (4)	C79—H79B	0.9900
C32—C37	1.404 (4)	C80—H80A	0.9900
C33—C34	1.394 (4)	C80—H80B	0.9900
C33—H33	0.9500	C81—C82	1.501 (4)
C34—C35	1.369 (5)	C81—H81A	0.9900
C34—H34	0.9500	C81—H81B	0.9900
C35—C36	1.383 (5)	C82—H82A	0.9900
C35—H35	0.9500	C82—H82B	0.9900
			
N1—Fe—N3	151.92 (8)	H41A—C41—H41B	109.5
N1—Fe—N4	87.11 (8)	C39—C41—H41C	109.5
N3—Fe—N4	86.61 (8)	H41A—C41—H41C	109.5
N1—Fe—N2	86.80 (8)	H41B—C41—H41C	109.5
N3—Fe—N2	85.43 (8)	C39—C42—H42A	109.5
N4—Fe—N2	150.72 (8)	C39—C42—H42B	109.5
N1—Fe—S	100.78 (6)	H42A—C42—H42B	109.5
N3—Fe—S	107.30 (6)	C39—C42—H42C	109.5
N4—Fe—S	103.24 (6)	H42A—C42—H42C	109.5
N2—Fe—S	106.03 (6)	H42B—C42—H42C	109.5
O6—K—O8	129.28 (7)	C44—C43—C48	119.3 (3)
O6—K—O7	93.90 (6)	C44—C43—C15	119.6 (3)
O8—K—O7	60.52 (6)	C48—C43—C15	120.7 (3)
O6—K—O10	97.01 (6)	C43—C44—C45	120.8 (3)
O8—K—O10	128.20 (6)	C43—C44—H44	119.6
O7—K—O10	99.85 (6)	C45—C44—H44	119.6
O6—K—O5	59.85 (6)	C46—C45—C44	119.8 (3)
O8—K—O5	94.90 (6)	C46—C45—H45	120.1
O7—K—O5	121.20 (6)	C44—C45—H45	120.1
O10—K—O5	132.29 (6)	C45—C46—C47	120.6 (3)
O6—K—O9	123.99 (7)	C45—C46—H46	119.7
O8—K—O9	100.69 (6)	C47—C46—H46	119.7
O7—K—O9	136.96 (6)	C46—C47—C48	119.9 (3)
O10—K—O9	59.65 (5)	C46—C47—H47	120.0
O5—K—O9	97.23 (6)	C48—C47—H47	120.0
O6—K—N9	120.80 (7)	C47—C48—C43	119.7 (3)
O8—K—N9	59.74 (7)	C47—C48—N7	123.2 (3)
O7—K—N9	120.11 (7)	C43—C48—N7	117.1 (2)
O10—K—N9	119.64 (6)	O3—C49—N7	122.4 (3)
O5—K—N9	61.11 (7)	O3—C49—C50	121.0 (3)
O9—K—N9	60.27 (6)	N7—C49—C50	116.6 (3)
O6—K—N10	59.46 (6)	C49—C50—C51	114.6 (3)
O8—K—N10	120.43 (6)	C49—C50—C53	106.9 (3)
O7—K—N10	60.09 (6)	C51—C50—C53	109.4 (3)
O10—K—N10	59.92 (6)	C49—C50—C52	108.2 (3)
O5—K—N10	119.18 (6)	C51—C50—C52	109.3 (3)
O9—K—N10	119.30 (6)	C53—C50—C52	108.2 (3)
N9—K—N10	179.56 (7)	C50—C51—H51A	109.5
C67—O5—C66	111.9 (2)	C50—C51—H51B	109.5
C67—O5—K	111.50 (17)	H51A—C51—H51B	109.5
C66—O5—K	112.12 (17)	C50—C51—H51C	109.5
C68—O6—C69	112.4 (2)	H51A—C51—H51C	109.5
C68—O6—K	118.13 (18)	H51B—C51—H51C	109.5
C69—O6—K	118.42 (17)	C50—C52—H52A	109.5
C72—O7—C73	111.1 (2)	C50—C52—H52B	109.5
C72—O7—K	112.16 (15)	H52A—C52—H52B	109.5
C73—O7—K	112.26 (17)	C50—C52—H52C	109.5
C75—O8—C74	113.2 (2)	H52A—C52—H52C	109.5
C75—O8—K	119.38 (18)	H52B—C52—H52C	109.5
C74—O8—K	115.16 (16)	C50—C53—H53A	109.5
C79—O9—C78	112.2 (2)	C50—C53—H53B	109.5
C79—O9—K	114.18 (16)	H53A—C53—H53B	109.5
C78—O9—K	114.18 (18)	C50—C53—H53C	109.5
C80—O10—C81	111.9 (2)	H53A—C53—H53C	109.5
C80—O10—K	114.30 (15)	H53B—C53—H53C	109.5
C81—O10—K	116.10 (16)	C55—C54—C59	117.8 (2)
C4—N1—C1	106.5 (2)	C55—C54—C20	119.7 (2)
C4—N1—Fe	127.09 (16)	C59—C54—C20	122.4 (2)
C1—N1—Fe	124.55 (16)	C56—C55—C54	122.4 (3)
C6—N2—C9	106.5 (2)	C56—C55—H55	118.8
C6—N2—Fe	126.43 (16)	C54—C55—H55	118.8
C9—N2—Fe	124.98 (16)	C55—C56—C57	118.7 (3)
C11—N3—C14	105.9 (2)	C55—C56—H56	120.7
C11—N3—Fe	125.25 (17)	C57—C56—H56	120.7
C14—N3—Fe	126.77 (16)	C58—C57—C56	120.8 (3)
C19—N4—C16	106.4 (2)	C58—C57—H57	119.6
C19—N4—Fe	124.68 (16)	C56—C57—H57	119.6
C16—N4—Fe	127.90 (16)	C57—C58—C59	120.5 (3)
C27—N5—C26	128.4 (2)	C57—C58—H58	119.7
C27—N5—HN5	115.8	C59—C58—H58	119.7
C26—N5—HN5	115.8	C58—C59—C54	119.8 (3)
C38—N6—C37	130.1 (3)	C58—C59—N8	121.9 (2)
C38—N6—HN6	114.9	C54—C59—N8	118.2 (2)
C37—N6—HN6	114.9	O4—C60—N8	121.4 (3)
C49—N7—C48	130.7 (2)	O4—C60—C61	120.4 (3)
C49—N7—HN7	114.6	N8—C60—C61	118.3 (3)
C48—N7—HN7	114.6	C62A—C61—C62	53.2 (11)
C60—N8—C59	127.6 (2)	C62A—C61—C60	111.9 (10)
C60—N8—HN8	116.2	C62—C61—C60	108.2 (3)
C59—N8—HN8	116.2	C62A—C61—C64	139.7 (10)
C65—N9—C77	109.9 (3)	C62—C61—C64	108.6 (4)
C65—N9—C76	110.3 (3)	C60—C61—C64	108.0 (3)
C77—N9—C76	109.8 (3)	C62A—C61—C63	55.9 (12)
C65—N9—K	107.88 (18)	C62—C61—C63	106.5 (4)
C77—N9—K	109.75 (18)	C60—C61—C63	112.9 (3)
C76—N9—K	109.17 (19)	C64—C61—C63	112.5 (4)
C70—N10—C71	109.9 (2)	C62A—C61—C63A	123.4 (15)
C70—N10—C82	110.5 (2)	C62—C61—C63A	141.4 (9)
C71—N10—C82	109.3 (2)	C60—C61—C63A	107.4 (9)
C70—N10—K	109.76 (16)	C64—C61—C63A	45.2 (10)
C71—N10—K	108.41 (16)	C63—C61—C63A	72.1 (11)
C82—N10—K	108.92 (16)	C62A—C61—C64A	115.5 (14)
N1—C1—C20	125.1 (2)	C62—C61—C64A	64.1 (9)
N1—C1—C2	109.4 (2)	C60—C61—C64A	99.3 (9)
C20—C1—C2	125.3 (2)	C64—C61—C64A	50.8 (9)
C3—C2—C1	107.4 (2)	C63—C61—C64A	147.6 (9)
C3—C2—H2	126.3	C63A—C61—C64A	95.9 (13)
C1—C2—H2	126.3	C61—C62—H62A	109.5
C2—C3—C4	106.8 (2)	C61—C62—H62B	109.5
C2—C3—H3	126.6	C61—C62—H62C	109.5
C4—C3—H3	126.6	C61—C63—H63A	109.5
N1—C4—C5	125.1 (2)	C61—C63—H63B	109.5
N1—C4—C3	109.9 (2)	C61—C63—H63C	109.5
C5—C4—C3	125.1 (2)	C61—C64—H64A	109.5
C6—C5—C4	125.8 (2)	C61—C64—H64B	109.5
C6—C5—C21	117.1 (2)	C61—C64—H64C	109.5
C4—C5—C21	117.1 (2)	C61—C62A—H62D	109.5
N2—C6—C5	125.5 (2)	C61—C62A—H62E	109.5
N2—C6—C7	109.6 (2)	H62D—C62A—H62E	109.5
C5—C6—C7	124.8 (2)	C61—C62A—H62F	109.5
C8—C7—C6	107.1 (2)	H62D—C62A—H62F	109.5
C8—C7—H7	126.4	H62E—C62A—H62F	109.5
C6—C7—H7	126.4	C61—C63A—H63D	109.5
C7—C8—C9	107.1 (2)	C61—C63A—H63E	109.5
C7—C8—H8	126.5	H63D—C63A—H63E	109.5
C9—C8—H8	126.5	C61—C63A—H63F	109.5
N2—C9—C10	124.9 (2)	H63D—C63A—H63F	109.5
N2—C9—C8	109.7 (2)	H63E—C63A—H63F	109.5
C10—C9—C8	125.3 (2)	C61—C64A—H64D	109.5
C9—C10—C11	125.5 (2)	C61—C64A—H64E	109.5
C9—C10—C32	117.8 (2)	H64D—C64A—H64E	109.5
C11—C10—C32	116.8 (2)	C61—C64A—H64F	109.5
N3—C11—C10	124.9 (2)	H64D—C64A—H64F	109.5
N3—C11—C12	110.2 (2)	H64E—C64A—H64F	109.5
C10—C11—C12	124.8 (2)	N9—C65—C66	113.5 (3)
C13—C12—C11	107.1 (2)	N9—C65—H65A	108.9
C13—C12—H12	126.5	C66—C65—H65A	108.9
C11—C12—H12	126.5	N9—C65—H65B	108.9
C12—C13—C14	107.2 (2)	C66—C65—H65B	108.9
C12—C13—H13	126.4	H65A—C65—H65B	107.7
C14—C13—H13	126.4	O5—C66—C65	109.3 (3)
N3—C14—C15	125.4 (2)	O5—C66—H66A	109.8
N3—C14—C13	109.6 (2)	C65—C66—H66A	109.8
C15—C14—C13	125.0 (2)	O5—C66—H66B	109.8
C16—C15—C14	125.8 (2)	C65—C66—H66B	109.8
C16—C15—C43	120.1 (2)	H66A—C66—H66B	108.3
C14—C15—C43	114.1 (2)	O5—C67—C68	109.9 (3)
N4—C16—C15	124.9 (2)	O5—C67—H67A	109.7
N4—C16—C17	109.7 (2)	C68—C67—H67A	109.7
C15—C16—C17	125.4 (2)	O5—C67—H67B	109.7
C18—C17—C16	107.1 (2)	C68—C67—H67B	109.7
C18—C17—H17	126.5	H67A—C67—H67B	108.2
C16—C17—H17	126.5	O6—C68—C67	108.6 (3)
C17—C18—C19	107.0 (2)	O6—C68—H68A	110.0
C17—C18—H18	126.5	C67—C68—H68A	110.0
C19—C18—H18	126.5	O6—C68—H68B	110.0
N4—C19—C20	125.0 (2)	C67—C68—H68B	110.0
N4—C19—C18	109.8 (2)	H68A—C68—H68B	108.4
C20—C19—C18	125.0 (2)	O6—C69—C70	109.1 (3)
C1—C20—C19	125.9 (2)	O6—C69—H69A	109.9
C1—C20—C54	115.7 (2)	C70—C69—H69A	109.9
C19—C20—C54	118.2 (2)	O6—C69—H69B	109.9
C22—C21—C26	118.8 (2)	C70—C69—H69B	109.9
C22—C21—C5	120.8 (2)	H69A—C69—H69B	108.3
C26—C21—C5	120.4 (2)	N10—C70—C69	113.4 (2)
C23—C22—C21	121.2 (3)	N10—C70—H70A	108.9
C23—C22—H22	119.4	C69—C70—H70A	108.9
C21—C22—H22	119.4	N10—C70—H70B	108.9
C22—C23—C24	119.3 (3)	C69—C70—H70B	108.9
C22—C23—H23	120.4	H70A—C70—H70B	107.7
C24—C23—H23	120.4	N10—C71—C72	112.8 (2)
C25—C24—C23	120.6 (3)	N10—C71—H71A	109.0
C25—C24—H24	119.7	C72—C71—H71A	109.0
C23—C24—H24	119.7	N10—C71—H71B	109.0
C24—C25—C26	120.2 (3)	C72—C71—H71B	109.0
C24—C25—H25	119.9	H71A—C71—H71B	107.8
C26—C25—H25	119.9	O7—C72—C71	108.2 (2)
C25—C26—C21	119.8 (3)	O7—C72—H72A	110.1
C25—C26—N5	122.6 (2)	C71—C72—H72A	110.1
C21—C26—N5	117.5 (2)	O7—C72—H72B	110.1
O1—C27—N5	121.7 (3)	C71—C72—H72B	110.1
O1—C27—C28	122.3 (3)	H72A—C72—H72B	108.4
N5—C27—C28	116.0 (3)	O7—C73—C74	109.8 (2)
C30—C28—C29	109.2 (3)	O7—C73—H73A	109.7
C30—C28—C31	108.8 (3)	C74—C73—H73A	109.7
C29—C28—C31	109.8 (3)	O7—C73—H73B	109.7
C30—C28—C27	113.9 (3)	C74—C73—H73B	109.7
C29—C28—C27	108.2 (3)	H73A—C73—H73B	108.2
C31—C28—C27	106.8 (3)	O8—C74—C73	108.9 (3)
C28—C29—H29A	109.5	O8—C74—H74A	109.9
C28—C29—H29B	109.5	C73—C74—H74A	109.9
H29A—C29—H29B	109.5	O8—C74—H74B	109.9
C28—C29—H29C	109.5	C73—C74—H74B	109.9
H29A—C29—H29C	109.5	H74A—C74—H74B	108.3
H29B—C29—H29C	109.5	O8—C75—C76	108.8 (3)
C28—C30—H30A	109.5	O8—C75—H75A	109.9
C28—C30—H30B	109.5	C76—C75—H75A	109.9
H30A—C30—H30B	109.5	O8—C75—H75B	109.9
C28—C30—H30C	109.5	C76—C75—H75B	109.9
H30A—C30—H30C	109.5	H75A—C75—H75B	108.3
H30B—C30—H30C	109.5	N9—C76—C75	113.5 (3)
C28—C31—H31A	109.5	N9—C76—H76A	108.9
C28—C31—H31B	109.5	C75—C76—H76A	108.9
H31A—C31—H31B	109.5	N9—C76—H76B	108.9
C28—C31—H31C	109.5	C75—C76—H76B	108.9
H31A—C31—H31C	109.5	H76A—C76—H76B	107.7
H31B—C31—H31C	109.5	N9—C77—C78	113.7 (3)
C33—C32—C37	119.0 (3)	N9—C77—H77A	108.8
C33—C32—C10	121.2 (3)	C78—C77—H77A	108.8
C37—C32—C10	119.7 (3)	N9—C77—H77B	108.8
C32—C33—C34	121.2 (3)	C78—C77—H77B	108.8
C32—C33—H33	119.4	H77A—C77—H77B	107.7
C34—C33—H33	119.4	O9—C78—C77	109.3 (3)
C35—C34—C33	119.0 (3)	O9—C78—H78A	109.8
C35—C34—H34	120.5	C77—C78—H78A	109.8
C33—C34—H34	120.5	O9—C78—H78B	109.8
C34—C35—C36	121.3 (3)	C77—C78—H78B	109.8
C34—C35—H35	119.4	H78A—C78—H78B	108.3
C36—C35—H35	119.4	O9—C79—C80	109.4 (2)
C35—C36—C37	119.7 (3)	O9—C79—H79A	109.8
C35—C36—H36	120.2	C80—C79—H79A	109.8
C37—C36—H36	120.2	O9—C79—H79B	109.8
C36—C37—C32	119.8 (3)	C80—C79—H79B	109.8
C36—C37—N6	123.3 (3)	H79A—C79—H79B	108.2
C32—C37—N6	116.9 (2)	O10—C80—C79	109.3 (2)
O2—C38—N6	121.3 (3)	O10—C80—H80A	109.8
O2—C38—C39	123.0 (3)	C79—C80—H80A	109.8
N6—C38—C39	115.6 (3)	O10—C80—H80B	109.8
C38—C39—C42	111.1 (3)	C79—C80—H80B	109.8
C38—C39—C40	107.9 (3)	H80A—C80—H80B	108.3
C42—C39—C40	109.0 (3)	O10—C81—C82	108.5 (2)
C38—C39—C41	109.4 (3)	O10—C81—H81A	110.0
C42—C39—C41	109.8 (3)	C82—C81—H81A	110.0
C40—C39—C41	109.6 (3)	O10—C81—H81B	110.0
C39—C40—H40A	109.5	C82—C81—H81B	110.0
C39—C40—H40B	109.5	H81A—C81—H81B	108.4
H40A—C40—H40B	109.5	N10—C82—C81	113.3 (2)
C39—C40—H40C	109.5	N10—C82—H82A	108.9
H40A—C40—H40C	109.5	C81—C82—H82A	108.9
H40B—C40—H40C	109.5	N10—C82—H82B	108.9
C39—C41—H41A	109.5	C81—C82—H82B	108.9
C39—C41—H41B	109.5	H82A—C82—H82B	107.7
